# Health and society in Chukotka: an overview

**DOI:** 10.3402/ijch.v72i0.20469

**Published:** 2013-03-19

**Authors:** Alexey A. Dudarev, Valery S. Chupakhin, Jon Øyvind Odland

**Affiliations:** 1 Northwest Public Health Research Centre, St. Petersburg, Russia; 2 AMAP Secretariat, University of Tromsø, Tromsø, Norway

**Keywords:** Chukotka, Russian Arctic, indigenous people, social-economic transition, healthcare, health, demography, mortality

## Abstract

This study provides a historical overview of the changes in the socio-economic and health status of the population of Chukotka, from the Soviet to the post-Soviet period, with special attention paid to the circumstances of indigenous people. Past health studies in Chukotka are reviewed and key demographic and health indicator data presented.

Since the 1990s, Chukotka's population has shrunk to a third of its former size due to emigration of non-indigenous and mostly younger people, with a corresponding increase in the mortality rate due to aging of the population. However, the indigenous population has remained stable. Among the most important causes of mortality are injuries. The living conditions of indigenous people continue to be a cause of concern, beset by high rates of poverty, unemployment, alcoholism, suicide and a variety of infectious diseases, such as tuberculosis and sexually transmitted infections. The economy, general infrastructure and health care system of Chukotka have been considerably improved by the Abramovich administration in the 2000s.

The Chukotka Autonomous Okrug is located on the north-eastern end of the Eurasia continent, as a wedge between the Pacific and Arctic Oceans (Fig. 1). It is surrounded by the East Siberian Sea, the Chukchi Sea and the Bering Sea. The region's area is 737,700 km^2^. Within the Russian Federation, it shares borders with the Republic of Sakha (Yakutiya), Magadan Oblast, and the former Koryak Autonomous Okrug. The Bering Strait separates Chukotka from Alaska. Half of Chukotka lies beyond the Arctic Circle.

**Fig. 1:**
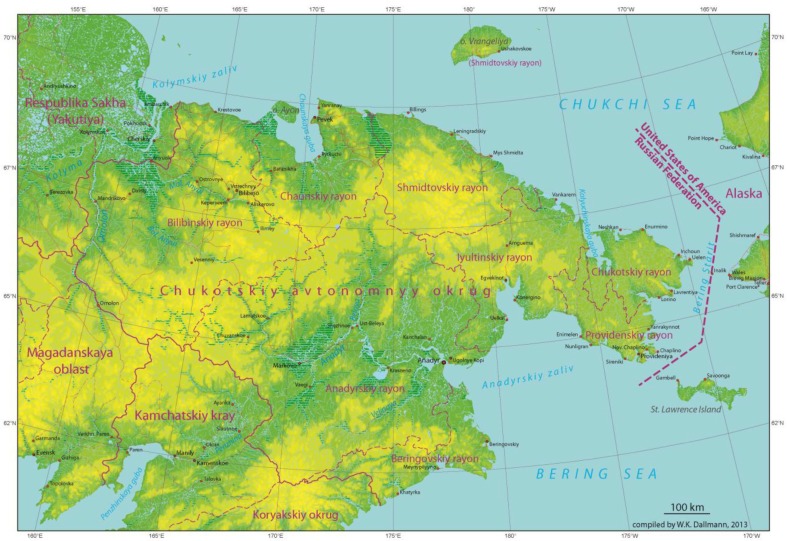
Map of Chukotka. Source: Winfried Dallmann, Norwegian Polar Institute, Tromsø.

The climate of Chukotka is determined by its geographical location in a zone influenced by 2 oceans, with a complex atmospheric movement involving cyclones of the European–Asian front, Arctic anti-cyclones and Southern cyclones. Chukotka has broken many climate records: maximum number of days without sun (Wrangel Island), highest average annual wind speed and highest frequency of snowstorms and hurricanes in Russia (Navarin Cape). Virtually every year, temperatures between −50°C and −60°C, and winds with speeds ranging from 50 to 60 m/sec are recorded. Permafrost extends from the upper layers of soil to a depth of tens and hundreds of metres. Within Chukotka are 3 cities (the capital Anadyr, Bilibino and Pevek), 15 townships and 45 villages in 6 administrative districts (Anadyrskiy, Bilibinskiy, Chaunskiy, Chukotskiy, Iultinskiy and Providenskiy). The total population of Chukotka in 2011 was 50,800, with a population density of 0.07 person/km^2^. About 16,000 persons were officially classified as “numerically small nationalities of the Russian North”, that is, indigenous people, among whom are Chukchi (75%), Eskimo (9%), Even (8%) and Chuvan (6%). During the past 20 years, the population of Chukotka has decreased by two-thirds due to emigration of non-indigenous people, including the families of military personnel.

Substantial literature on Chukotka's history, politics and economics is now available in English, focusing on research in the first decade of the post-Soviet era (1). This article provides an update on more recent developments, especially during and after the governorship of Abramovich since 2002.

## Regional economic development

The gross regional product per capita in Chukotka today is the 4th highest among the regions of the Russian Federation, after the oil-extracting Tyumen and Sakhalin regions, and Moscow (2). Chukotka has an affluent resource base, high-capacity energy sources and, in the Soviet period, a stable management and supply system. The territory of Chukotka contains 10% of the known gold deposits of Russia; there are also rich minefields of tin, copper, tungsten, platinum, silver, coal, oil and gas. Bilibino Nuclear Power Station is one of the two power stations in Russia located beyond the Arctic Circle, though it supplies electricity only to the mining centres nearby. Other areas depend on locally produced coal or imported fuel oil for their energy needs.

### The Soviet period

Economic development of Chukotka began during the period 1928–1936. The Central Board of the Northern Sea Route and Chukotka Aviation were organised; sea ports, airports, polar stations and industrial enterprises were constructed, and extensive hydrographic and geological surveys began. In 1934–1937, the Peveksky and Iultinsky tin–tungsten deposits were discovered. From 1939, prospecting works were conducted by NKVD Dalstroy, a special construction branch of the People's Commissariat of Internal Affairs, forerunner of the KGB. A series of forced labour camps – part of the GULag system – was established in the Kolyma region (composed of Chukotka and Magadan Oblast) to develop the region. GULag in Russian is the acronym for the “Chief Administration of Corrective Labour Camps and Colonies”.

The policies of forced collectivisation of indigenous people and “liquidation” of the kulaks (rich peasants) were implemented. Reindeer breeding, fishery and hunting of sea mammals grew rapidly. The fishing industry passed from coastal fishing to open sea trawling. During the same period, the written Chukchi and Eskimo languages were developed, illiteracy drastically reduced, resulting in the appearance of indigenous intelligentsia.

During World War II, substantial deposits of gold and other metals were discovered and coal mining also began. Chukotka was key to the war effort, as it provided not only metals for the army's needs but also furs, venison, fish and other necessities.

In the post-war years, the Kolyma region was designated by Stalin as an “industrial complex of special function”. The Dalstroy system lasted until the 1950s, and camps began to be closed after Stalin's death. During the Cold War, air-rocket units of the Soviet armed forces were located in Chukotka, increasing the number of frontier posts.

In 1958, gold was first extracted on an industrial scale. The 1960s underwent a period of intense construction activity – new mines, ore processing and other industrial enterprises, power stations, electric power transmission lines, roads, settlements, schools, kindergartens, resorts for workers, and so on. Manufacture of building materials started successfully. The Bilibinskaya nuclear power station, and the Chaunskaya and Ekvekinotskaya fuel-burning power stations transformed Chukotka into a net exporter of electric power. This decade also witnessed the maximum harvesting of reindeers, sea mammals, fish and furs. Dairy animal farming, swine breeding, greenhousing and fur farming were launched.

In 1915, there was only one elementary school in Chukotka; by 1977 there were 96 secondary schools and some technical schools; more than 11,000 children were cared for in 112 preschool institutions; 88 libraries and 106 cultural centres flourished.

Due to the northern bonus, the salaries in Chukotka appeared to be considerably higher than in other arctic regions of the USSR and much higher than the national average. As a result, Chukotka was well supplied with qualified professionals and the population grew due to the influx of immigrants. The state guaranteed certain privileges for the indigenous people, such as child benefits, free medicine, pregnancy care, and university entrance all over the USSR without competition.

### Post-Soviet period

With the collapse of the Soviet Union in 1991, Chukotka suffered a period of severe economic decline and depopulation. Under the governorship (1991–1999) of Alexander Nazarov, the region became deserted, scores of settlements were abandoned, including military sites. Construction came to a standstill; many indigenous villages had not been renovated since the 1950s and looked dilapidated. The mining industry had collapsed, gold mining was no longer considered profitable, unemployment was rampant, and fuel and food supplies were scarce. There were acute food shortages. The “Great Depression” had begun. The population was teetering on the edge of survival. The “Russian market revolution” for Chukotka had turned into a catastrophe.

Reindeer breeding was drastically reduced. The once-flourishing hunting and fur trade was in decline, while poaching was on the rise. Health care and education were in a poor state: Chukotka hospitals did not have enough X-ray machines, and schools did not have enough textbooks. Because of rampant alcoholism and other diseases, Chukotka natives were on the verge of ruin.

Things began to change in January 2001, when Roman Abramovich was appointed by the Kremlin as governor of Chukotka. Abramovich, a billionaire, the owner of Sibneft Oil Company and, since 2003, the owner of Chelsea Football Club in England, was one of Russia's richest oligarchs. When Abramovich arrived, Chukotka was bankrupt with huge external debts equivalent to 4 times its annual budgets, and state employees’ salaries had not been paid for half a year. The state together with the Savings Bank disappeared from Chukotka rapidly at the beginning of 1990s. Money left Chukotka as promptly as the people left. Of about 160,000 residents of Chukotka at the end of the 1980s, only 70,000 people remained at the beginning of the new millennium.

At first, in 2001, Abramovich organised humanitarian help – each resident received 20 kg of sugar, 5 kg of salt, a sack of flour, a sack of potatoes, 5 kg of butter and 5 kg of dried fruits. Abramovich registered 3 branches of Sibneft Company in Chukotka. Simply by moving to the region, Abramovich added some tens of millions of dollars in tax revenues to Chukotka's meagre budget. Perhaps for the first time in the recent history of Russia, money had come to the region instead of leaving. Builders from Turkey and Canada came to the region and large-scale construction began across Chukotka – building and renovation of houses, schools, roads, boiler-houses, heating systems and bakeries.

Within a few years, the region was thoroughly renovated and modernised. The old Soviet dreariness was gone from the capital Anadyr, which now boasts a new airport, roads, hotels, an orthodox church, college, university centre, hospital, sport-fitness centre, supermarket and automatic teller machines.

Between 2001 and 2008, 100,000 square metres of housing, 18 new schools, 28 hospitals and medical centres were put into operation. Hot water, flush toilet, and double-glazed windows were obligatory in the new houses. The majority of houses were painted in pretty blue, red and yellow colours.

The system of financing and management was rejuvenated, and the delivery of goods from European Russia by the Northern Sea Route was resumed. Products and essential commodities again appeared in shops after an absence of 8 years. Abramovich also made sure that the salaries of public sector workers were paid on time.

Children in schools received new computers, televisions, textbooks, free food and free health services. The Internet was available in many schools. The Chukchi language and culture, which only recently were threatened with extinction, were reintroduced to the school curriculum. Each year, all children (about 6,000) were sent on a free summer vacation by the Black Sea.

Gold mining began operating on a modern technological basis, and oil exploration and extraction started in the continental shelf offshore.

Sea mammal hunting brigades in the coastal indigenous communities were supplied with big, new metal hunting boats equipped with powerful Japanese engines. Reindeer breeding was re-developed in the inland communities.

The Chukchi revered Abramovich as the source of many good things; some even considered him a deity to be prayed to. He was feted with concerts and performances.

Yet this “paradise” was temporary and mostly superficial. Many old problems persisted. The most qualified, skilled and enterprising workers had already left the region. Abramovich's reforms were confronted with the indifference and apathy of many people who had lost the desire and ability to work. Few wanted to solve the problems themselves, instead waited for outside help – from the state in the past, and now from Abramovich.

Abramovich decreed the upper limit of Chukotka's population to be between 40,000 and 45,000, a number deemed to be financially sustainable by the region's resources. People who did not want to work were encouraged to leave Chukotka for other parts of Russia where they were provided with housing and allowance for 3 years. For people who remained in Chukotka, the administration tried to inculcate market values and methods, but much of the population had become accustomed to dependence the old fashioned way.

Abramovich resigned from the position of governor in July 2008, while officially retaining the chairmanship of the Chukotka Duma (regional assembly). He was replaced as governor by his deputy Roman Kopin.

Overall, the economic crisis experienced by Chukotka during the 1990s had been successfully overcome by the regional administration during the governorship of Abramovich. Presently, the climate for investment in Chukotka remains attractive, while the economy and population have stabilised.

## Situation of indigenous people

The 2 major groups of indigenous people in Chukotka are the Chukchi and the Eskimos (3). Their population has remained relatively stable during the 20th century, fluctuating between 11,000 and 13,000 Chukchi, and between 1,000 and 1,500 Eskimos.

According to the 2002 census, 15,767 Chukchi lived in the Russian Federation, of whom 12,622 persons (about 70%) lived in Chukotka. Chukchi live in all settlements of Chukotka together with Russians, Eskimo, Evens and other people. There are no exclusively Chukchi settlements, although the Chukchi constitute the majority group in most of the villages. Today, the Chukchi are mainly engaged in commercial reindeer breeding. Chukotka is the only region in the world where stock of domestic reindeer is growing, but it is much less than it was during the Soviet period. While reindeer breeding is a collective occupation, fishing and hunting are common as personal and family activities of the indigenous population.

Eskimo live in the eastern coastal settlements of Chukotka. There are about 1,450 Eskimos today. Almost 80% of the Eskimo population is concentrated in Providensky and Chukotskiy districts, especially in 2 villages – Novoje Chaplino and Sireniki. Relatively large Eskimo communities can also be found in 5 villages – Provideniya, Lavrentija, Lorino, Uelkal and Uelen. The proportion of mixed marriages of Eskimo (with Russians and Chukchi) varies from 40 to 65%. The current distribution of the Eskimo population is the result of large-scale forced relocations of some 800 Eskimos in 1958, about 70% of the population, leaving behind some of the previously large villages, such as Naukan and Chaplino, uninhabited (4).

The basic traditional employment of Eskimo and coastal Chukchi is sea mammal hunting – walrus, seal and whale. Whale hunting is conducted today strictly according to internationally agreed quota: for Russia the International Whaling Commission has established a quota of 135 grey and 5 Greenlandic whales (in 2010).

A part of the population lives in hardly accessible remote villages dependent on transportation, which is at times unpredictable. Transportation in Chukotka is characterised by the absence of railways and roads (due to permafrost), and 90% of freight is moved by sea and air (2).

Despite Abramovich's reforms, the socio-economic conditions in many areas of Chukotka are still difficult, beset by problems of supplying settlements with fuel, foodstuff and electricity. Many settlements endure poor water supply, sanitation and housing. Outside Anadyr, the land still needs a major clean-up. Many coastal settlements and their vicinities are littered with hundreds of thousands of abandoned rusty fuel barrels, scrap metal and other junk. Solid waste pollution is less of a problem in the inland villages.

Poverty and unemployment among indigenous people promotes a host of poor health and social conditions. Underdevelopment of public health services and general infrastructure in indigenous villages have resulted in high infant and general mortality, and a high burden of infectious diseases, such as tuberculosis and sexually transmitted diseases (5).

The survival of indigenous languages is precarious. Total Russification of school education in the Soviet period, when children were in essence forbidden to use their native languages in schools, has led to the situation today where Chukchi and Eskimo languages are considered native to only one-third of the indigenous people. The Sireniki Eskimo dialect has totally disappeared. Now, efforts for reviving the Chukchi language are undertaken – it is taught in many settlements, and it is included in the high school programme. Chukchi language is widely used in art, political literature and the mass media (6).

## Health care delivery

Chukotka has a centralised model of health care services. In 2002, all treatment and preventive establishments were reorganised into a network consisting of the Chukotka Okrug Hospital in Anadyr, 5 regional hospitals, 19 local hospitals, 6 medical ambulance stations and 15 medical-obstetric aid units. Patients in far flung reindeer-breeding brigades and remote villages are evacuated by air to the closest regional centre, and this is highly dependent on the weather.

Between 2001 and 2011, 15 new health care facilities have been constructed and 27 renovated. The Chukotka Okrug Hospital is equipped with modern radiological and ultrasonographic devices and other medical–surgical equipment.

Health care financing, since the middle of 2007, is under a model of single payer through the local fund of obligatory medical insurance (OMI). Per capita health care expenditure in 2010 was 37,700 rubles, the highest in the Russian Federation.

In 2009, in Chukotka, 313 doctors were employed (61.6 doctors per 10,000 people), which was substantially below the 575 doctors deemed to be needed for the region. Today the administration is planning to implement measures to attract physicians to Chukotka.

Thirteen pharmacies operate in Chukotka – 6 governmental, 2 municipal and 5 private. There is no wholesale trade in medical products. The private sector accounts for less than 50% of the pharmaceutical market. Delivery of medical products is carried out independently by pharmaceutical institutions by air or sea from Vladivostok. The remote, sparsely populated settlements are supplied by the governmental and municipal pharmacies under contracts with local hospitals, ambulance stations and medical-obstetric aid units.

## Health research

Up to the mid-1960s, the Russian Arctic, particularly Chukotka, had the status of a secret zone due to its strategic economic and defence importance. There were multiple military installations (such as submarine and icebreaker fleets), nuclear processing and testing sites, rocket-firing grounds, gold–diamond, oil–gas and other mining operations, and an extensive network of prison camps (the gulag). Almost all scientific information connected with the Arctic (including health studies) was strictly prohibited from publication in the open literature; even dissertations were defended in “special” scientific councils. During the 1970s and 1980s, the situation improved marginally but the prevailing communist ideology was to suppress unwelcome information, such as the poor health conditions of the indigenous people.

Since the end of 1980s, political freedom slowly reached even the Russian Arctic. Nevertheless, even today, the Chukotka Autonomous Okrug is still considered a frontier regime, and even Russian citizens are obliged to apply for special permission to enter its territory. The severe economic decline of the post-Soviet period (described above) also affected the conduct of health research, which was fragmentary and uncoordinated. Help came from abroad. Agencies, such as the University of Alaska, the United States Centres for Disease Control and the Alaska Native Medical Centre, were able to collaborate with the Siberian branch of the Russian Academy of Medical Sciences. Collaborative projects, such as the Alaska Siberia Medical Research Program (7), investigating a wide variety of health topics, such as alcoholism, genetics, immunity, nutrition, diabetes and cardiovascular diseases, were established. The history and output of this collaboration has recently been reviewed (8). The increasing use of market foods and decreasing consumption of traditional foods among Chukotka native adults was found to contribute to the increasing burden of obesity, diabetes mellitus and cancer (9), although the prevalence of diagnosed diabetes among Chukchi and Eskimo was much lower than in indigenous populations in Alaska and northern Canada (10). Few cases of diabetes, thyrotoxicosis and obesity were detected in a review of morbidity among native children in 1993–1997, which the authors attributed to underdiagnosis due to the lack of specialised laboratories and personnel in Chukotka (11). Also, the prevalence of a variety of infectious diseases (such as chronic otitis, chronic bronchitis and pneumonia), mental disorders and poisoning by drugs and toxic substances was several times higher than in the rest of Russia (11). An assessment of maternal and children health care in Chukotka in 1997–2000 revealed that the proportion of completely healthy children decreased 10 times during the decade, from 25% to only 2%; a high rate of premature births as well as high child and maternal mortality were also noted (12).

Analysis of tuberculosis in Chukotka in 1994–1999 suggests that despite economic difficulties, there was a continuous reduction in the major tuberculosis indicators; this may be accounted for by inadequate detection of tuberculosis among natives and by low contacts with newcomers (13). Among zoonoses, brucellosis has been shown to be endemic in about 50% of reindeer farms, putting reindeer breeders and workers at risk (14, 15). Sexually transmitted diseases are rampant among the indigenous population, particularly gonorrhea, which is 3–6 times higher than in the whole of Russia. In 1998, 3 cases of AIDS were recorded for the first time.

Because of changing nutritional pattern of indigenous people towards increasing fish consumption (also fresh-frozen “stroganina”), they are at risk of exposure to a variety of parasitic diseases. The true prevalence is likely much higher than officially documented, as few surveys had been conducted among indigenous people living in remote settlements and tundra brigades. Almost 100% of common fish species (keta, gorbusha, nerka, whitefish, char and smelt) are contaminated by several types of parasites, among them *Diphyllobotrium*, *Corynosoma* and some nematodes. Surveys among children have found infection rates of enterobiasis as high as 23%, and diphyllobothriasis in 13–34% of the examinees (16).

Echinococcosis is endemic in Chukotka. Trichinosis is also widespread and widely distributed among wildlife, such as bears (100%), wolves (57%), foxes (37%), Arctic foxes (15%), and also domestic cats (10%) and dogs (5%) (15). Inadequate sanitary living conditions and close co-habitation with animals promote the dissemination of many infectious and parasitic diseases.

The economic collapse of the infrastructure of Chukotka region in the early post-Soviet years resulted in many indigenous people reverting to the traditional subsistence economy. Relatively expensive market foods were replaced by cheaper ones, and by more readily available local foods. The per cent contribution of proteins, lipids and carbohydrates to total caloric intake did not change substantially, but the sources of the major nutrients were different from Soviet times. In 1985, local marine mammals accounted for about half of the consumed meat (55%), while in 2000 its share increased to 89%. Market fats and oils were substituted by the fat of marine mammals (17, 18). More recent changes in the diet of the people remain to be documented, and their long-term impact of health needs to be monitored.

## Review of health and demographic indicators

### Sources of statistical data

Official health statistics on mortality and morbidity of the population of the various administrative territories in the Russian Federation, and earlier in the RSFSR (Russian Soviet Federative Socialist Republic within the USSR), was never detailed and systematised due to the absence of registries. Some small, special regional registries, such as cancer (Arkhangelsk Oblast), births (Murmansk Oblast) or occupational diseases (some Siberian regions), were only established in the first decade of the 21st century, the first 2 with the help of Norway (19, 20).

The collection of health statistics on Russian Arctic regions such as Chukotka is complicated by the frequent redrawing of the political map. Chukotka Okrug became autonomous in 1980 but as part of Magadan oblast. In 1992, it became a separate “subject of the Russian Federation” (i.e. with status equal to that of a republic, kray and oblast, sending separate elected representatives to the federal Duma or parliament). Earlier in the Soviet period, between 1930 and 1980, Chukotka was subordinated variously to the Far East Kray, Kamchatka Oblast, or Khabarovsk Kray. Official state statistics on Chukotka were not available in published yearbooks and other state statistical documents, until the 1990s.

For the period 1976–1985, some health statistics on the general Chukotka population have been collected by the Chukotka Okrug Hospital (Anadyr) Medical Statistics Bureau in a series of working papers (21–25). These cover population, fertility, mortality and morbidity, with some years providing specific data on indigenous people. The first official statistical publication on Chukotka (26) covers the period 2004–2008, produced by the Chukotka Statistical Agency.

Another source of data is the Russian Demographic Yearbooks (27–29), both for the Chukotka general population and the whole of the RSFSR or Russian Federation serving as a comparison. Additional data fragments are scattered in various documents of Goskomstat (Russian federal and USSR statistics).

### Statistical data on indigenous people

There is no unified state system of health monitoring of indigenous people in the Russian Federation. While a Federal Law of 1999 guarantees the rights of indigenous numerically small nationalities of the Russian Federation, how an indigenous person is defined is unclear. In the 2002 Russian census, a person can declare himself to belong to any nationality. There is no longer any section for “nationality” or “ethnicity” in Russian passports. In 1989, there were 128 distinct “nationalities” in the entire USSR; today there are 150 in Russia alone. In the 2002 census, 68 newly self-designated ethnic categories appeared. The 2010 census was widely criticised as poorly conducted, and detailed results have not been released. Under the circumstances of modern Russian national policy, when Arctic territories are under extensive exploitation for natural resources, the problems of indigenous health are very far from the priorities of the government.

In the Soviet period, within the territories of indigenous people, personal ethnic identity was recorded in several documents (birth and death data records, passports, community “economy books”, etc.). It was possible to separate out demographic and limited health data pertaining to indigenous minorities in locally produced statistics. Since 2002, such information is no longer available.

### Demographic characteristics

**Fig. 2:**
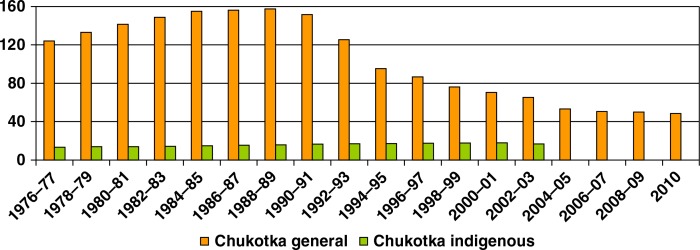
Total and indigenous population of Chukotka (1,000 persons), 1976–2010. Note: Information on indigenous population after 2002 not available.

As mentioned in the introduction, the population of Chukotka by the mid-2000s increased to about a third of what it was in the 1980s (Fig. 2), mostly due to mass emigration of non-indigenous people, while the size of the indigenous population has increased from 13,300 in the 1970s to 17,900 at the beginning of the 2000s (30).

Table I shows the size and composition of the indigenous population of Chukotka. It is characterised by the predominance of Chukchi (70–82%); followed by the Eskimo (8–9%); and smaller number of Koryaks, Yukagirs and Evens.

**Table I:** Population of indigenous people in the Chukotka Autonomous Okrug, 1970–2002

	1970		1979		1989		2002	
				
	n	%	n	%	n	%	n	%
Total	13,382		13,883		15,903		17,938	
Chukchi	11,001	82.2	11,292	81.3	11,914	74.9	12,619	70.3
Eskimo	1,149	8.6	1,278	9.2	1,452	9.1	1,534	8.6
Evens	1,061	7.9	1,077	7.8	1,336	8.4	886	4.9
Others	171	1.3	236	1.7	1,201	7.6	2,899	16.2

**Fig. 3:**
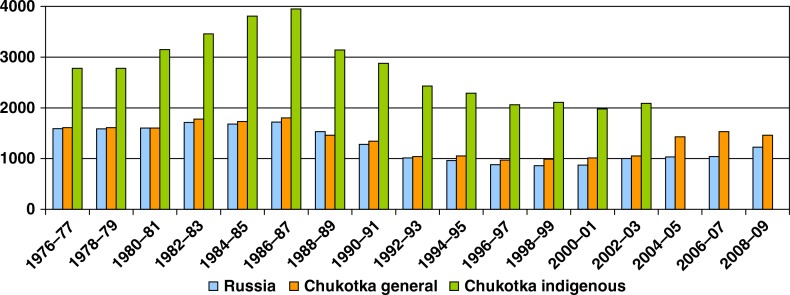
Birth rates in Chukotka and Russia (per 100,000), 1976–2009. Note: Information on indigenous population after 2002 not available.

Birth rates among Chukotka indigenous people were much higher (on average: 1.5–2 times) than among the general population of Chukotka and Russia (Fig. 3).

**Fig. 4:**
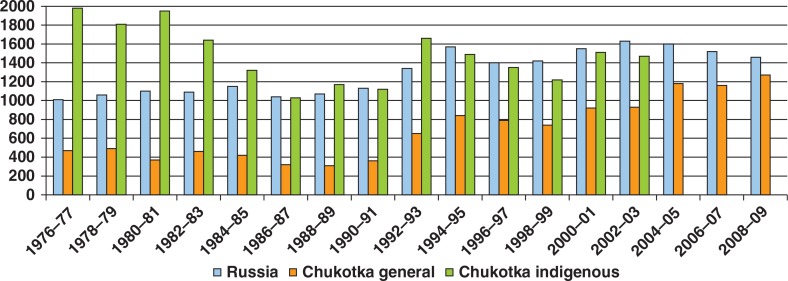
Crude mortality rates (all causes mortality) in Chukotka and Russia (per 100,000), 1976–2009. Note: Information on indigenous population after 2002 not available.

The crude mortality rate among the general population of Chukotka was about half that of Russia as a whole, up to the mid-1990s (Fig. 4). However, since the 2000s, it has increased significantly due to ageing of the population caused by out-migration of younger people. Mortality among the indigenous population in Chukotka was very high in the 1970s and 1980s (4 times higher than among the general Chukotka population and 1.5–2 times higher than in Russia). By the mid-1990s, it was comparable to the Russian rate but still 1.5 times higher than among the general Chukotka population.

**Fig. 5:**
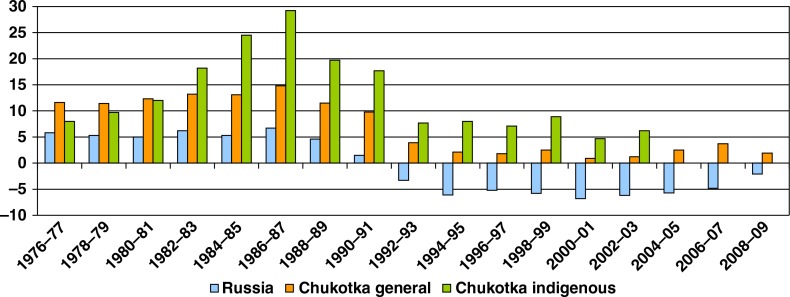
Natural increase of population in Chukotka (per 1,000), 1976–2009. Note: Information on indigenous population after 2002 not available.

The natural increase in the general population of Chukotka was twice that in Russia during the 1970s and 1980s, and it sharply dropped in the 1990s, although remaining slightly positive (i.e. more births than deaths). Nationally, since the breakup of the USSR, Russia had experienced high mortality and low fertility, a situation that verges on demographic collapse. A natural increase in the indigenous population of Chukotka had consistently been higher than in either Russia or Chukotka as a whole. It also suffered a sharp decrease in the 1990s but was able to remain positive (Fig. 5).

### Causes of mortality

**Fig. 6:**
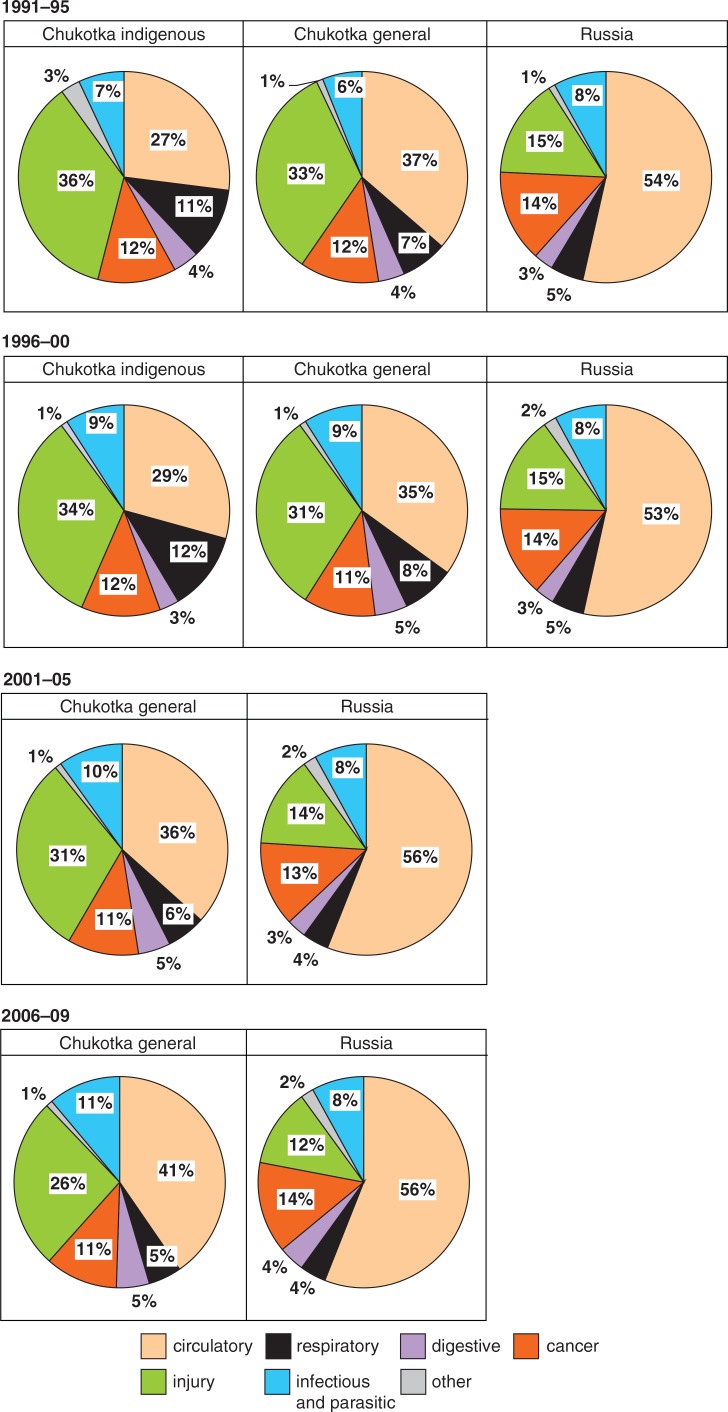
Distribution of causes of mortality among the indigenous and general populations of Chukotka and Russia, 1991–2009. Note: Information on indigenous population after 2002 not available.

The causes of mortality among Chukotka natives have remained stable: injuries account for one-third of the total number of deaths, compared to only 15% in Russia. Ranked second is cardiovascular diseases (Fig. 6). The mortality experience of the general population of Chukotka mirrors that of its native population, with a slightly higher proportion attributed to cardiovascular diseases and a lower proportion to injury.

## Conclusions

Chukotka underwent massive social and economic changes since the breakup of the Soviet Union, although the economy has rebounded and infrastructure has improved during the Abramovich governorship of 2001–2008. Since the 1990s Chukotka's population has shrunk to a third of its former size due to emigration of non-indigenous and mostly younger people, with a corresponding increase in the mortality rate due to aging of the population. However, the indigenous population has remained stable. Among the most important causes of mortality are injuries. The living conditions of the indigenous people continue to be a cause of concern, beset by high rates of poverty, unemployment, alcoholism, suicide and a variety of infectious diseases, such as tuberculosis and sexually transmitted infections. The economy, general infrastructure and healthcare system of Chukotka have been considerably improved by the Abramovich administration in the 2000s.
